# Visualizing Cross-Sections of 3D Objects: Developing Efficient Measures Using Item Response Theory

**DOI:** 10.3390/jintelligence11110205

**Published:** 2023-10-28

**Authors:** Mitchell E. Munns, Chuanxiuyue He, Alexis Topete, Mary Hegarty

**Affiliations:** Department of Psychological & Brain Sciences, University of California, Santa Barbara, CA 93106, USAhegarty@ucsb.edu (M.H.)

**Keywords:** spatial ability, cross-section, STEM education, item response theory, personnel selection

## Abstract

Spatial ability is important for success in STEM fields but is typically measured using a small number of tests that were not developed in the STEM context, have not been normed with recent samples, or have not been subjected to modern psychometric analyses. Here, an approach to developing valid, reliable, and efficient computer-based tests of spatial skills is proposed and illustrated via the development of an efficient test of the ability to visualize cross-sections of three-dimensional (3D) objects. After pilot testing, three measures of this ability were administered online to 498 participants (256 females, aged 18–20). Two of the measures, the Santa Barbara Solids and Planes of Reference tests had good psychometric properties and measured a domain-general ability to visualize cross-sections, with sub-factors related to item difficulty. Item-level statistics informed the development of the refined versions of these tests and a combined measure composed of the most informative test items. Sex and ethnicity had no significant effects on the combined measure after controlling for mathematics education, verbal ability, and age. The measures ofcross-sectioning ability developed in the context of geology education were found to be too difficult, likely because they measured domain knowledge in addition to cross-sectioning ability. Recommendations are made for the use of cross-section tests in selection and training and for the more general development of spatial ability measures.

## 1. Introduction

Since the early 20th century, measures of spatial intelligence have been used in selection for technically demanding jobs, such as mechanic and pilot (Smith 1964). More recently, studies have shown that spatial intelligence is predictive of success in science, technology, engineering, and mathematics (STEM) education, even after controlling for individual differences in verbal and mathematical ability (Shea et al. 2001; Wai et al. 2009), and that spatial skills can be improved with various types of training (Uttal et al. 2013). As a result of these developments, there has been a groundswell of interest among educators and psychologists in the possibility of improving STEM outcomes by fostering the development of spatial skills. To study this prospect, we need valid and reliable assessments of STEM-relevant spatial abilities and skills, which are normed for the relevant student and professional populations. However, many existing tests of spatial ability were not informed by STEM education and have not been adequately validated or normed. Here, we describe an approach to developing valid, reliable, and efficient computer-based tests of spatial skills and norming them on representative samples of the population using modern psychometric techniques, including Item Response Theory (IRT). We illustrate this approach by developing an efficient test of the STEM-relevant ability to visualize a cross-section of a three-dimensional (3D) object, examining possible sub-components of this ability and assessing how demographic variables affect performance on this measure.

There is no shortage of spatial ability tests. In their International Directory of Spatial Tests, Eliot and Smith (1983) listed 392 different spatial tests. Unfortunately, no agreement has been reached on the classification of these tests. Historically, approaches to classifying spatial tests relied on exploratory factor analysis (e.g., Michael et al. 1957; McGee 1979; Lohman 1988; Carroll 1993) or on task analysis (Linn and Petersen 1985). However, the factor analysis results differed, depending on the specific tests included in the battery and the statistical techniques used, and the field never arrived at a consensus based on these studies. In fact, only a handful of the many tests, notably tests of mental rotation, spatial visualization, and perspective taking, are in common use (Hegarty 2018; Malanchini et al. 2020), likely because they are more openly available for research use.

Existing tests of spatial ability were developed in the early-to-mid 20th century to select personnel for technical occupations (Smith 1964; Hegarty and Waller 2005). However, as they were not developed in the context of STEM education, it is not clear that they measure the most relevant spatial skills for STEM learning. For example, recent studies have identified the importance of spatial cognitive tasks that are important for success in STEM, such as imagining non-rigid transformations (Atit et al. 2013) and cross-sections of three-dimensional structures (Kali and Orion 1996), but were not measured using existing tests or included in classic factor analytic studies. Researchers have developed their own tests of these abilities but, in most cases, have not subjected them to full psychometric analyses.

There are a number of methodological limitations to the current state of spatial ability testing. First, even the classic tests of spatial ability that are commonly in use have not been normed on recent cohorts, while more recently developed tests have typically been normed on small convenience samples of college students, if at all. Second, most spatial tests have not been subjected to modern psychometric analyses such as Item Response Theory (IRT), which allows for examining the reliability and validity of individual test items, enabling the identification of the most informative items for measuring the relevant ability. Third, researchers have adapted existing tests by shortening them for efficient measurement or converting them to online measures without any examination of the psychometric properties of the adapted tests. As a result, we currently have many tests that purportedly measure the same cognitive process (e.g., mental rotation, cross-sectioning, perspective taking) but may actually tap different capabilities, whereas tests with different names may tap the same ability (Brucato et al. 2022). For all of these reasons, we do not know enough about what current tests measure to make effective recommendations regarding which students might need educational interventions to succeed in STEM education or technical careers or to evaluate the effects of interventions that aim to improve spatial thinking.

**Figure 1:**
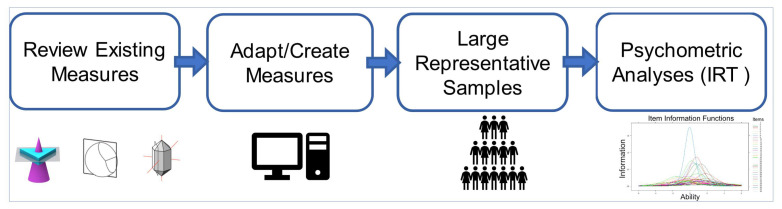
Schematic of our approach to developing and standardizing measures of spatial ability.

Here, we propose and demonstrate an approach to address these problems (see Figure 1) by developing robust and efficient measures of STEM-relevant spatial abilities. The first step is to identify spatial skills that we know are related to STEM success and to review existing measures of each skill.

The second step is to adapt these measures for online testing, which, in turn, enables the collection of large samples of data representing the population at large. Most current tests exist only in paper-and-pencil forms. Because many of these tests have a time limit, individuals with lower spatial abilities typically do not complete all of the items on these tests, which has hampered the application of modern psychometric analyses, such as IRT, to these tests. Applying these techniques requires large data sets (typically of 500 or more participants), in addition to requiring responses to all test items for all participants. Adapting these tests for computer-based administration facilitates the collection of large data sets and can enable us to impose a time limit per item rather than for a whole test or subtest so that we collect data on all items from all participants.

The third step is to collect data on these tests from large samples that are representative of the general population. As noted, existing tests of spatial abilities have not been normed, or if they have, it has typically been on convenience samples of college students. Many technical careers require more practical experience or technical training rather than a college education, so it is important to examine skills across the whole adult population, and not just those who go to college. It is also important to ensure that our samples reflect the ethnic and socio-economic diversity of the population. 

The fourth and final step is to subject the data to statistical analyses. First, we need to examine the internal consistency and correlations between the tests, as measures of reliability and validity. As some measures were developed for specific populations, it is also important to examine the relative difficulty of their items for the general population. Item Response Theory (IRT, Hambleton et al. 1991) can be valuable in accomplishing these goals. IRT analysis involves fitting a model to observed responses on each item of a test based on the probability of a test taker at a given ability level getting that item correct. It offers several advantages over the classical test theory (Hambleton and Jones 1993). First, estimated item parameters are sample independent so that the results of an IRT analysis are more accurate when generalizing to larger populations. Second, IRT provides a measure of precision, which allows the researcher to examine how precisely a test can measure the construct at different levels of ability. For example, a test item may offer a precise measure at a high level of ability but be less precise at lower ability levels. Using IRT, we can identify the most discriminating and precise items from different tests, enabling the development of efficient tests. 

Our approach was informed by a recent study on the spatial perspective-taking ability. Brucato et al. (2022) conducted an analysis of four measures in common use to measure this ability. Correlational and IRT analyses revealed that although the four measures ostensibly measure the same ability, one test was not significantly correlated with the other three, suggesting that it measured a somewhat different ability. The other tests differed in characteristics, such as whether the display included a human figure or three-dimensional cues, but the tests measured a common ability. Item Response Theory analysis was used to identify the most discriminating items on these tests and also indicated that they differed in discriminability across the range of ability. This analysis was used to recommend a set of items that could be used to create efficient and reliable measures of perspective-taking ability. This study shows the promise of aspects of our approach but used existing paper-and-pencil measures and was limited by a relatively small sample and a college student sample. Here, we use the same approach to examine cross-sectioning tests, first adapting them for online administration. This enabled us to recruit a national sample of 18–20-year-olds, including high school students, college students, and individuals not currently in an educational institution. 

### 1.1. Cross-Sectioning 

We focus on the spatial task of inferring the cross-sections of a 3D object. While this task was not included in classic factor analyses of spatial ability measures (e.g., Michael et al. 1957; McGee 1979; Lohman 1988; Carroll 1993), it has been identified as a spatial skill that is central to spatial thinking in science, technology, engineering, and mathematics. A cross-section is the resulting plane or flat surface after a three-dimensional object is cut using a two-dimensional plane, that is, a 2D slice of a 3D object. Cross-sections are often used in science and technology to show the internal structure of a three-dimensional object, such as a complex mechanical system or a part of the human anatomy. They are also observed in everyday activities such as cooking (slicing fruits and vegetables), and as a result, the development of cross-sectioning skills might not be dependent on formal education. 

Tests of cross-sectioning have been related to achievement in biology, medicine, geology, engineering, and geometry. In biology, they are commonly used to mentally represent cross-sections of anatomical structures, and this skill is also important in interpreting medical images, such as X-rays, ultrasounds, and magnetic resonance imaging (Hegarty et al. 2007). Rochford (1985) found that medical students who performed poorly on sectioning geometric solids also performed poorly (compared to high-spatial students) on practical anatomy exams. Russell-Gebbett (1985) found a similar pattern with younger students (age 11 to 14); specifically, students’ science ability (as rated by their teachers) correlated positively with their performance on Biology test items that required them to imagine a cross-section of a three-dimensional structure. In geology, students need to be able to visualize the internal structure of a geological formation (Kali and Orion 1996). Engineering also draws heavily on this skill, specifically when working with blueprints and making orthographic projections (Duesbury and O’Neil 1996; Gerson et al. 2001; Hsi et al. 1997). Finally, Pittalis and Christou (2010) identified skills such as the understanding of 2D representations of 3D objects as being a predictive factor of 3D geometric reasoning abilities. Overall, the ability to visualize cross-sections is important for success in a range of STEM fields. 

**Figure 2:**
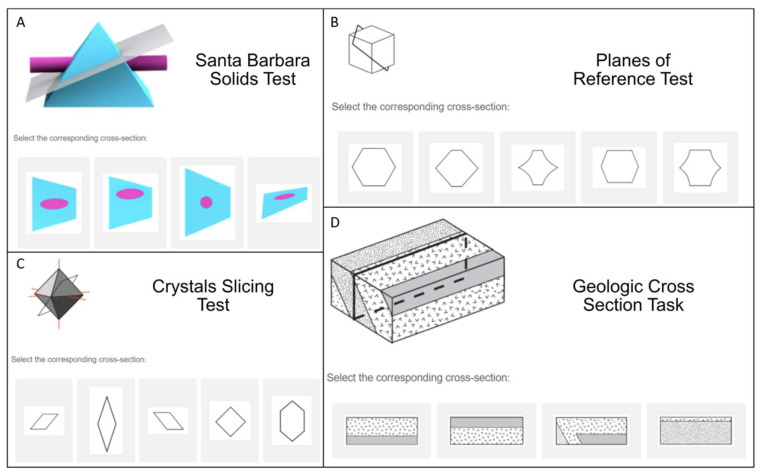
Sample items from the four cross-section tests used in this research. (**A**) Santa Barbara Solids Test (SBST) item (**B**) Crystal Slicing Test (CST) item (**C**) Planes of Reference Test (PRT) item (**D**) Geologic Block Cross-Sectioning Test (GBCST) item.

Several tests are currently in use to measure cross-sectioning ability with no agreed-upon standard. The earliest test of this ability is the Mental Cutting Test (MCT, College Entrance Examination Board 1939), which has 25 items that show a representation of a 3D shape with a plane cutting through it and requires test takers to choose the answer that shows the shape of the cutting plane (see Figure 2B for an example). This test is very similar to another commonly used cross-section test called the Planes of Reference test (PRT, Titus and Horsman 2009), a 15-item test with the same instructions as the MCT but with distinct items. In both of these, the figures are shown as line drawings with limited depth cues. Another test, also referred to as the Mental Cutting Test or “Schnitte” (Quaiser-Pohl 2003), shows similar figures to the MCT but uses a different method of responding (“select all that apply” rather than choosing one correct answer out of five choices). These tests likely involve similar spatial visualization processes but afford different analytic strategies due to the response formats. 

Cohen and Hegarty (2007) developed the Santa Barbara Solids Test (SBST), with the same multiple-choice format as the original Mental Cutting Test and Planes of Reference test but different stimuli that were rendered with 3D imaging software and included shading to provide depth cues (see Figure 2A). The items were designed to vary in difficulty based on two aspects: the geometric complexity of the solid and the orientation of the cutting plane. Finally, a number of tests of cross-sectioning ability were designed for studies of specific STEM disciplines. Some examples include the Crystal Slicing Test (CST, Ormand et al. 2017), the Geologic Block Cross-Sectioning test (GBCST, Ormand et al. 2014) designed for testing Geology students, and the tooth cross-section test (Hegarty et al. 2009) designed for testing dental students. The Crystal Slicing Test (see Figure 2C) shows 3D crystals found in mineralogy, while the Geologic Block Cross-Sectioning Test (See Figure 2D) shows sections via geological structures. The tooth cross-section task uses 3D figures of a tooth with roots inside and, like the Geologic Block Cross-Sectioning test, involves visualizing internal structure, so these tests are often referred to as tests of penetrative thinking (Kali and Orion 1996). 

### 1.2. This Present Study 

This present study began by comparing performance on four of the existing cross-section tests: the Santa Barbara Solids Test (Cohen and Hegarty 2007), the Planes of Reference Test (Titus and Horsman 2009), the Crystal Slicing Test (Ormand et al. 2017), and the Geologic Block Cross-Sectioning test (Ormand et al. 2014; see examples of test items in Figure 1)^1^. First, these tests were adapted for online administration via Qualtrics. After piloting the online tests with a college sample, we removed the Geologic Block Cross-Sectioning test from further analysis due to its difficulty and time constraints and administered the remaining three tests in an online study. This enabled us to collect a large data set necessary to conduct Item Response Theory analyses from a sample that is more representative of the US population. 

One goal of the research was to examine the psychometric properties of existing tests. We first examined the internal consistency of these tests and their inter-correlations to assess their reliability and validity and whether they measure a common ability despite the differences between the tests (e.g., 3D depth cues, type of structure to be sectioned, etc.). Next, we applied IRT analysis to establish whether items on each test measure a common ability or capture unique variance and to assess the difficulty and discriminability of the items on each test. This enabled us to construct an efficient measure of cross-sectioning, made up of the most discriminating items on the existing tests, a second goal of this research.

Using this refined test, we then considered different models of the cognitive processes underlying the cross-sectioning ability by examining possible subcomponents of this ability and aspects of items that affect their relative difficulty. First, we considered the orientation of the cutting plane. Previous research with the Santa Barbara Solids Test indicated that people scored lower on items with cutting planes that are oblique to the reference frame of the object than for orthogonal cutting planes (Cohen and Hegarty 2007, 2012), possibly because people have more experience with near-orthogonal cuts in everyday life (e.g., when slicing vegetables). Although it is unlikely that these everyday cross-sections are perfectly orthogonal, they are generally close to shapes created by an exact 90° cut. Oblique cuts might also be more difficult to identify correctly because of a tendency to infer that a 2D shape would extend orthogonally into 3D space, not obliquely, and so we might tend to visualize a rectangle as an orthogonal cut of a rectangular prism instead of an oblique cut of a cube (Gagnier and Shipley 2016). Similarly, using the Mental Cutting Test, Tsutsumi (2004, p. 117) identified two types of items: “pattern” problems, which only require recognizing the shape of the cut to solve it (e.g., a rectangle), and “quantity” problems, which also require identifying metric properties of the shape (e.g., the aspect ratio of the correct shape). Second, we considered the complexity of the solid, including the number of parts making up the solid, which has also been found to affect difficulty (Cohen and Hegarty 2007, 2012). Therefore, we also considered models that took complexity into account. 

Finally, we examined how demographic variables (including sex, age, ethnicity, and mathematics education) are related to cross-sectioning performance. Sex differences are found in some but not all spatial ability measures (Linn and Petersen 1985; Voyer et al. 1995), so it is important to establish whether these differences exist (and the size of any differences) for different spatial measures. To our knowledge, this is the first study to examine sex differences in cross-section tests with a large sample. In contrast to sex differences, there has been relatively little research on the relationship between ethnicity and spatial performance. Here, we compared the performance of Hispanic and non-Hispanic participants. Because cross-sections are studied to some extent in geometry and cross-section diagrams are prevalent in scientific textbooks, it is also possible that this spatial skill is affected by education, so we compared the performance of individuals with different levels of education (e.g., high school vs. college) and explored the effects of parental education and taking math courses.

### 1.3. Study 1: Pilot with University Students

A pilot study was conducted with university students to assess the feasibility of administering cross-section tests online, to measure the average time taken to respond to items in the cross-section tests, and to establish basic levels of performance for a general college population on the four tests.

## 2. Materials and Methods

### 2.1. Participants

Participants were 45 students in introductory psychology courses who received course credit for participation. Data from two participants were removed because they took a very long time to complete the test (more than 100 min; the mean time for other subjects was 26.53 min, *SD* = 13.12 min). Sex of students was not recorded.

### 2.2. Materials

The following four tests were adapted from their original paper and pencil versions and administered online via Qualtrics.

#### 2.2.1. Santa Barbara Solids Test (SBST, Cohen and Hegarty 2012)

The Santa Barbara Solids Test is a multiple-choice test on the ability to imagine cross-sections of solid objects. It contains 30 items varying in complexity of the solids, from simple solids (e.g., a cube) to objects made up of connected solids or embedded solids (see sample item in Figure 1). With respect to complexity, 10 items present a geometric primitive (cone, cube, cylinder, prism, or pyramid) as the solid, 10 items show objects made up of two geometric primitives joined at an edge, and 10 items are made up of two primitives, with one embedded inside the other. The orientation of the cutting planes is varied, with half of the cutting planes being orthogonal to the main axis of the solid while the others are oblique. There are 4 possible answer choices for each item, so chance performance (30/4) is 7.5. 

#### 2.2.2. Planes of Reference Test (PRT, Titus and Horsman 2009)

In this test, participants are asked to choose the shape of the intersection of a slicing plane with a geometric solid. The solids are represented as line drawings with no shading (see Figure 1). There are 15 items on the test, and each item is worth one point, with no penalty for incorrect answers. There are 5 possible answer choices for each item, so chance performance (15/5) is 3.0.

#### 2.2.3. Crystal Slicing Test (CST, Ormand et al. 2017)

In the Crystal Slicing Test, participants choose which shape would be made by slicing a geometric solid with a plane from five answer choices. The solids are symmetrical across the x (lateral) and z (depth) axes and have shading to provide depth cues (see Figure 1). The solids are the shapes of common crystals in mineralogy. There are 15 items on the test. Each item is worth one point, and there is no penalty for incorrect answers. There are 5 possible answer choices for each item, so chance performance (15/5) is 3.0.

#### 2.2.4. Geologic Block Cross-Sectioning Test (GBCST, Ormand et al. 2014)

This multiple-choice test requires students to select the cross-section produced using a pictured cut through a Geologic Block Diagram (see example stimulus in Figure 1 below). This test was developed for geology majors and has been used primarily in studies related to spatial ability in geosciences (Gold et al. 2018; Hannula 2019; Ormand et al. 2014). It has 16 items; each item is worth one point, and there is no penalty for incorrect answers. There are 4 possible answer choices for each item, so chance performance (16/4) is 4.0.

### 2.3. Procedure

This study was implemented online via Qualtrics. After giving informed consent, students were administered the four tests, which were preceded by standard instructions for these tests. Both the order of tests and the order of items within the tests were randomized. Test items were presented one at a time. Response time was measured for each item, and there was no time limit. 

## 3. Results

Descriptive statistics and measures of reliability for the four tests are presented in Table 1. McDonald’s Omega measures general factor saturation. Internal consistency was calculated using Spearman–Brown (Spearman 1904) corrected split-half reliability using the ‘splithalf’ package in R (Parsons et al. 2019) and Cronbach’s Alpha. Mean performance on the Santa Barbara Solids Test was well above chance (95% CI [17.26, 21.39], chance = 7.5), and this test also showed good reliability. The performance of the Planes of Reference (95% CI [6.38, 8.23], chance = 3) and Crystal Slicing (95% CI [7.39, 9.12], chance = 3) tests were also well above chance, but these tests showed only moderate reliability. The mean performance of the Geologic Block Cross-Sectioning Test was significantly above but close to chance (95% CI [4.87, 6.66], chance = 4), and this test also showed moderate reliability.

Response times per item were similar for the SBST, PRT, and CST (see Table 1). The median response times for these tests were 10.46, 10.78, and 11.43, respectively, and only five participants had median response times of more than 20 s per item on any of these tests. In contrast, the mean (see Table 1) and median (16.88) response times for the GBCST were substantially longer, and 31.1% of response times were more than 20 s. As shown in Table 2, correlations between the tests were high, especially after correcting for the reliability of the measures, suggesting that they share considerable variance. 

## 4. Discussion

The pilot study indicated that the Santa Barbara Solids Test, the Planes of Reference Test, and the Crystal Slicing Test were of appropriate difficulty for college students, while the Geologic Block Cross-Sectioning test was more difficult and also took more time per item. While these results should be interpreted with caution, due to the small sample size in this study, it is notable that performance on the SBST was tested close to chance, although significantly greater than chance, when administered without a time limit. In particular, 37.2% of college students were at or below chance performance on the GBCST, while the percentage of students at or below chance on the other tests was below 12%. It is likely that the GBCST test depends on geology knowledge in addition to the ability to imagine cross-sections. Ormand et al. (2014) describe the test as “…a geoscience-specific test of penetrative thinking…” (p. 149), and previous research indicates that expert geologists score better on this test than college students (Tarampi et al. 2016) and that students perform better on this test after a geology course (Hannula 2019). It is also the only test of the four that involves complex internal structures, as the stimuli in the other tests are made up of regular solids. Because of its possible reliance on geology knowledge, we anticipated that it might be more difficult for the general population, who are less likely to have this knowledge. For these reasons and the need to keep the overall time requirement manageable for online administration, the GBCST was not included in the large online study. Instead, it would be valuable to compare the GBCST to other tests involving internal structures or geology-specific knowledge in future research, as it possibly captures a somewhat separable component of cross-section ability. Based on the response times observed in the pilot study, participants were given a time limit of 20 sec for each item in the large online study. 

### Study 2: Large Representative Sample

**Figure 3:**
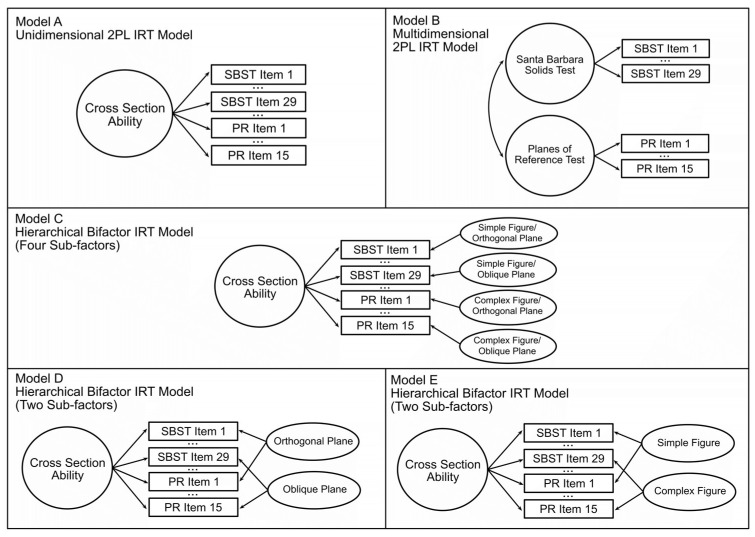
Possible unidimensional, multidimensional, and hierarchical models of cross-sectioning ability.

In Study 2, we administered the Santa Barbara Solids, Planes of Reference, and Crystal Slicing tests online to a large sample representative of the US population. This enabled us to use the IRT analyses to examine whether these tests measure one unitary construct and test for item-specific factors. To first examine the psychometric properties of the individual tests, we fit a unidimensional, two-parameter logistic model (2PL) to each of the three tests to assess the model fit and the difficulty and discriminability of test items. Then, we developed an efficient, hybrid test of cross-sectioning ability that functions well over a wide range of abilities. This included items from the SBST and PRT tests. We then fit another unidimensional, 2PL model to this test (Model A, See Figure 3) and compared it to a multidimensional 2PL model (Model B) in which the SBST and PRT items were assumed to measure distinct abilities in order to investigate whether these tests measure the same ability or separate, correlated abilities. A series of hierarchical bifactor models were evaluated in order to examine if the tests measure a general cross-section ability with correlated sub-abilities that explain the variance between different types of items. Different combinations of sub-abilities were tested (Models C, D, and E) based on previously observed difficulty factors, i.e., the angle of the cutting plane (orthogonal vs. oblique) and complexity of the solid to be sectioned (Cohen and Hegarty 2007, 2012). Finally, the relations between demographic variables (sex, race, ethnicity, and education) and performance on this refined measure were examined. 

## 5. Materials and Methods

### 5.1. Participants

Five hundred and twelve participants (260 female) aged 18–20 were recruited from Qualtrics Panels, met Qualtrics’ automatic screening process, and were paid for their time. Twelve participants’ data were removed due to timing out (leading to missing data on at least 20% of the items), one participant was removed for answering ‘C’ to all but one item, and one was removed for scoring zero on two of three cross-section tests, leaving a total of 498 participants in the analysis. Samples of 500 have been suggested to accurately estimate IRT parameters for a test with 20 items, and the minimum recommended sample decreases with more test items (Sahin and Anil 2017). The demographics of the sample, which were chosen to match the racial breakdown of Navy recruits^2^, are presented in Table 3. Participants were drawn from all geographic regions (n = 132 Northeast, n = 88 Southwest, n = 80 West, n = 116 Southeast, and n = 82 Midwest) and environment (n = 181 cities, n = 202 suburbs, and n = 115 small town/rural) to ensure that data were collected from all areas of the US. The majority of the participants were native English speakers (n = 472).

### 5.2. Materials

Participants were administered the Santa Barbara Solids Test (Cohen and Hegarty 2012), Crystal Slicing Test (Ormand et al. 2017), and the Planes of Reference Test (Titus and Horsman 2009). The tests were identical to those used in the pilot study except that the instructions included a sample item that participants were required to answer correctly before proceeding to the test items, were required to spend at least five seconds on each item, and were given a time limit of 20 s for each item. Participants also completed the Wordsum Plus Vocabulary Test of the General Social Survey (Cor et al. 2012), a 14-item version of the General Social Survey’s vocabulary test (Word Sum), which added 4 additional items to the original 10-item test. The added words were chosen to provide more information in the moderate ability range based on IRT analysis. 

### 5.3. Procedure

All tests were completed online either using a desktop computer or a mobile phone. They were completed in one session, which took approximately 25 min. Participants first provided informed consent, followed by a warning message explaining that the tests would require attention and effort and that their data would be unusable if they did not try their best. The warning was used as an attempt to improve data quality by making an appeal to conscience as well as preventing attrition due to the difficulty of the tests (Zhou and Fishbach 2016).

Participants completed the three cross-section tests in a random order. Each test was preceded by standard instructions that could not be advanced until a certain amount of time had expired (10–15 s, depending on the length of the page) to encourage participants to read the instructions completely. Then, each test included one sample problem, which participants had to answer correctly before proceeding to the test items (they were given multiple attempts at this item if necessary). The order of items in each test was randomized. Participants were required to spend a minimum of 5 s on each item and were allowed a maximum of 20 s. After completing all three cross-section tests, participants completed the Word Sum test. Lastly, participants were given an attention check (Aust et al. 2013) asking if they had made a serious attempt. 

## 6. Results

### 6.1. Scoring

Correct answers were coded as 1, and incorrect answers were coded as 0. If a participant reached the time limit of 20 s for a problem without selecting an answer choice, they were assigned a score of 0 for that problem. The time limit was reached with no answer selected on a mean of 3.2% of items. 

### 6.2. Descriptive Statistics

**Figure 4:**
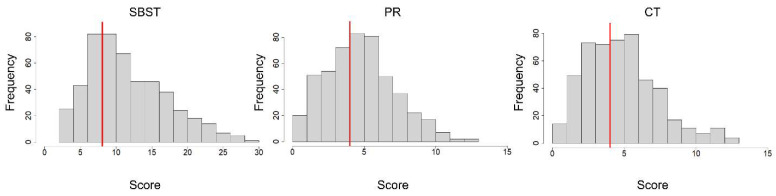
Distribution of scores on each cross-section test. Santa Barbara Solids Tests (SBST), Planes of Reference (PRT), Crystal Slicing Tests (CST) frequency of scores. The vertical red line indicates chance performance.

Descriptive statistics for the measures are shown in Table 4, and histograms showing their distributions are in Figure 4. The average scores on the three cross-section tests were above chance levels (chance was 7.5 for the SBST and 3.0 for both the PRT and CST). However, they were closer to chance than for the college student sample in Study 1. The average scores were 12.10 on the SBST, 5.24 on the PRT, and 5.28 on the CST. The average response times for the cross-section tests (see Table 4) were well within the imposed time limit of 20 s. The performance of the Word Sum test was also above the chance level (2.8) and was not timed in this administration. 

### 6.3. Reliability and Correlations

Internal consistency and general factor saturation (McDonald’s Omega) for the four measures are shown in Table 4. Internal consistency was calculated using Spearman–Brown (Spearman 1904) corrected split-half reliability using the ‘splithalf’ package in R (Parsons et al. 2019) and Cronbach’s Alpha. Like in Study 1, the Santa Barbara Solids Test showed good reliability, while the reliability of the Planes of Reference and Crystal Slicing Tests was moderate. As shown in Table 5, correlations between the tests were moderate and remained significant after controlling for verbal ability (Word Sum test), indicating that, as expected, the cross-section tests shared common variance related to spatial processing and not just motivation or general intelligence (providing evidence for divergent validity). The disattenuated correlations between the measures were also calculated, taking their reliabilities (i.e., permutation-based split-half estimation) into account (Parsons et al. 2019). The disattenuated correlations between the cross-section measures were high (0.90 or greater), indicating considerable common variance. Disattenuated correlations are reported above the diagonal in Table 5. 

### 6.4. Unidimensionality and Local Independence

**Figure 5:**
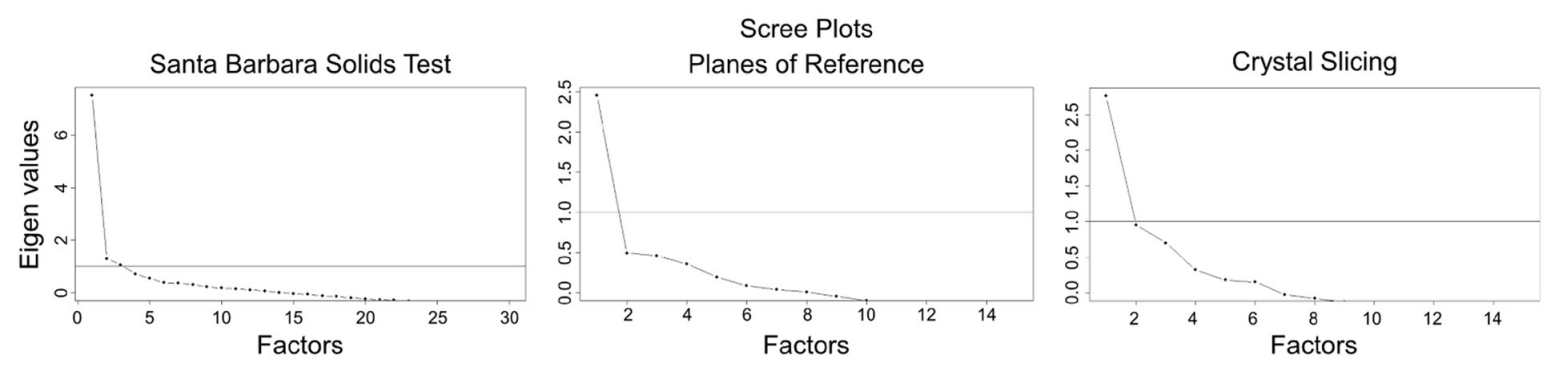
Scree plots for the Santa Barbara Solids Test, the Planes of Reference Test, and the Crystal Slicing Test.

We conducted an exploratory factor analysis from the tetrachoric correlation matrices to assess whether each of the cross-section tests measured a unidimensional ability, using two criteria to assess the number of factors (dimensions) underlying performance, the number of factors with Eigenvalues greater than 1, and the Scree test (Cattell 1966). The first factor in the SBST had an Eigenvalue of 7.52, while the second and third Eigenvalues were just over 1 (1.29 and 1.05, respectively). The first factor was clearly dominant, and the scree plot suggested one factor (see Figure 5). The PRT also showed a dominant first factor (first Eigenvalue = 2.45, second Eigenvalue = 0.49), as did the CST (first Eigenvalue = 2.76, second Eigenvalue = 0.95), and for these tests, only Eigenvalues of the first factor exceeded 1, again suggesting unidimensionality.

The tests were also assessed for local independence of items. If a person’s score on one item predicts their score on another item after accounting for the main factor of cross-section ability, the pair of items is said to be locally dependent and likely share a common feature separate from cross-sectioning. To test for local dependence, the G^2^ statistic (Chen and Thissen 1997) and the Jackknife Slope Index (JSI; Edwards et al. 2018) were calculated for each possible combination of items in each individual test using the *residuals* function in the R package *mirt* (Chalmers 2012). In order to examine each model for possible locally dependent item pairs, the distribution of the standardized values of G^2^ and JSI for all item pairs was inspected (see the range for each diagnostic in Table 6). There was no evidence of local dependent item pairs.

### 6.5. Within Task Item Response Theory Analyses

We tested the fit of the data for each test to the single-factor, two-parameter logistic model (2PL), which includes estimated parameters for discriminability and difficulty for the test items. All models were fit using the “mirt” package in R (Chalmers 2012) using the Expectation Maximization (EM) algorithm (Bock and Aitkin 1981). Table 7 presents the model fit for each test. The overall model fit for each test was assessed using the M_2_ statistic, which has been demonstrated to be less influenced by the sparsity of the contingency table of response patterns than chi-square (Maydeu-Olivares 2014), as well as Root Mean Square Error of Approximation (RMSEA), Comparative Fit Index (CFI), Tucker–Lewis Index (TLI), and Standardized Root Mean Squared Residual (SRMR). Acceptable fit was determined based on the following criteria: Root Mean Square Error of Approximation (RMSEA) below 0.05 (Maydeu-Olivares 2013), Comparative Fit Index (CFI) above 0.95, Tucker–Lewis Index (TLI) above 0.95^3^, and SRMR less than 0.05 (Maydeu-Olivares 2013). While a significant M_2_ indicates that we should reject the null hypothesis of the fitted model being true, this statistic was small relative to the degrees of freedom ($\frac{M_{2}}{df}$ < 3), indicating an acceptable model fit.

### 6.6. Santa Barbara Solids Test

For the SBST, the unidimensional 2PL model is not a good fit based on all model fit diagnostics, but most were close to the cutoffs. The individual items of SBST were also examined to check for sources of model misfit. A well-fitting item will have a non-significant p-value in a test of chi-square, indicating that the model’s generated data were not significantly different from the observed data. Item 8 showed marginally significant misfit (*p* = .025). However, the RMSEA of this item is 0.038, which is still well within the acceptable range (<0.05). Removing item 8 and re-running the model results in another item being significantly misfit, and this repeats if the next item is also removed. As such, the overall model fit does not improve by removing item 8, and it was maintained. 

**Figure 6:**
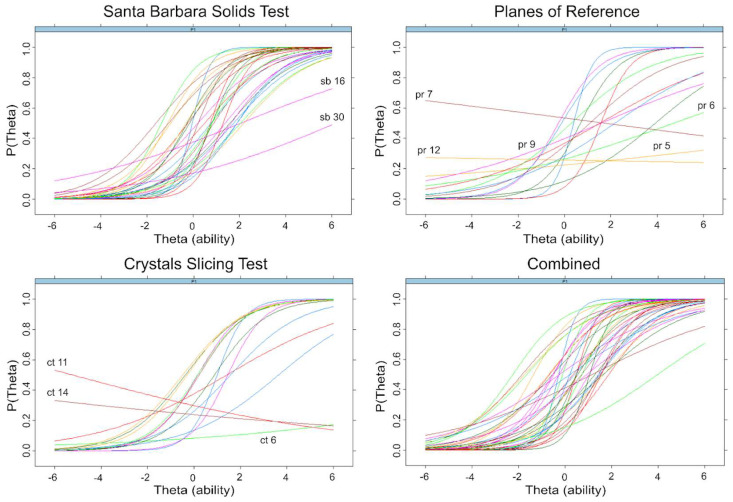
Item characteristic curves for the Santa Barbara Solids Test (SBST), Planes of Reference (PRT), Crystal Slicing Test, and a combined test consisting of the 38 discriminating items on the SBST and PRT. P(Theta) is the probability of getting each item (represented by different colored lines) correct at a given ability level (Theta). Items with low discriminability are labeled for SBST, PRT, and CST. The combined test is the refined SBST and PRT.

Item characteristic curves (ICC) shown in Figure 6 visualize the estimated parameters of discriminability and difficulty for each item of the tests (See Supplementary Table S1 for all SBST item parameters). The slope of each ICC represents the discriminability of one item. If an ICC for a given item is flat (i.e., has a low slope), this indicates low discriminability such that at low levels of ability (x-axis), the probability of getting the item correct (y-axis) is not much lower than at high levels of ability. The location of an ICC on the x-axis indicates its difficulty, with items to the right indicating more difficulty. 

The mean discriminability coefficient for all 30 SBST items was 0.98 (moderate), and they ranged from 0.25 (very low) to 2.58 (very high). As seen in Figure 4, items 16 and 30 do not discriminate well between high and low levels of ability. The discriminability coefficients of these items were less than or equal to 0.26. In both of these items, there were two answer choices that were very similar. These items were removed to improve the efficiency of the test. The mean discriminability coefficient for the remaining 28 items was 1.04 and ranged from 0.67 to 2.59. 

The mean of the difficulty coefficients for the SBST was −0.49, meaning the items were somewhat difficult (a test-taker with average ability would have a less than 50% chance of getting an item correct on average). The item difficulties ranged from −2.12 (very difficult) to 1.73 (very easy). After removing items 16 and 30, the average difficulty coefficient was −0.46, and the range did not change. 

**Figure 7:**
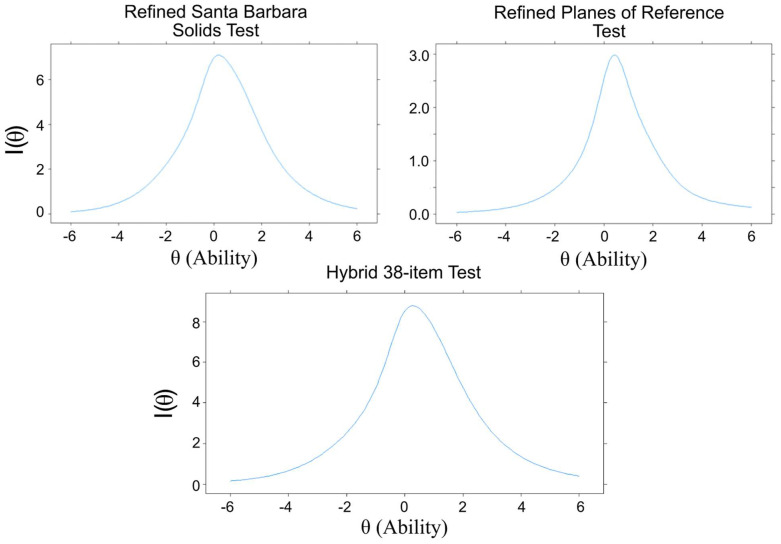
Test information functions for refined versions of SBST, PRT, and the 38-item test with SBST and PRT Items. I(θ) is the information that the test gives (i.e., how precise the θ estimate is) at a given θ (level of ability).

The Test Information Function (TIF, Figure 7) shows how much information the overall tests capture at a given level of ability. For SBST, the curve of the TIF was highest around average to slightly above-average ability (theta = 0 to 0.5), meaning the test is more precise when measuring test takers in this ability range. Overall, the test should perform well on test takers between moderately low (theta = −1) and high (theta = 1.5) ability levels, with the most precision around average (theta = 0) ability. 

### 6.7. Planes of Reference Test

For the Planes of Reference Test, the unidimensional 2PL model fits acceptably by all criteria (see Table 7). The fit of individual items was assessed using a signed chi-square, and there were two marginally misfit items (item 2, *p* = 0.022, and item 9, *p* = 0.039). As with the SBST, these items had acceptable RMSEAs at 0.05 or less. The model fit did not improve with the removal of these items, and they were maintained in the refined version of the test.

The average discriminability for all PRT items was 0.68, and it ranged from −0.08 (very low) to 2.45 (very high). The average difficulty was −0.78 (moderately difficult) and ranged from −2.45 (very difficult) to 0.29 (average). All items with a discriminability coefficient less than 0.26 (items 5, 6, 7, 9, and 12) were removed in order to improve test efficiency, leaving 10 items. The discriminability coefficients of these 10 items ranged from 0.35 to 2.45. The TIF of the overall PRT test (Figure 4) shows the most precision for test takers of average to above-average ability (thetas from 0 to 1). See Supplementary Table S2 for all PRT item parameters.

### 6.8. Crystal Slicing

Overall, the Crystal Slicing Test did not show an acceptable model fit. The RMSEA of the model was acceptable, but the CFI, TLI, and M_2_ significance tests indicated a poor fit (see Table 7). The consideration of individual items indicated that items 2, 10, and 14 showed significant misfits (*p* < .05). Item 10 had the highest RMSEA (0.081), while the other two were within acceptable bounds (≤0.05). While there were not any obvious problems with these items upon inspection, one possible source of error for non-geology students might have misinterpreted the symmetry of the shapes as they are unlikely to be familiar with these shapes. 

After removing items 2, 10, and 14 and fitting a new 2PL model, the model fit improved but still did not fit acceptably (*p* < .001, RMSEA = 0.049, CFI 0.85, TLI 0.82). The removal of three more items (1, 6, 9) that have significant misfits in the new model (*p* < .05) indicated a poorer fit of the overall model. The remaining nine items did not show any individual misfits. Thus, removing any more items would not improve fit. In sum, the 2PL model does not fit the observed data for the Crystal Slicing Test, and as a result, it was not included in the analysis of the combined tests.

### 6.9. Combined Task Item Response Theory Analysis: Model Comparisons

A refined 38-item cross-section test was created comprising the 28 SBST and 10 PRT items that showed good discriminability. The SBST images of the solids to be sliced had depth cues, including shading, while the PRT items showed line drawings with limited depth cues (see Figure 2) and, therefore, may have depended more on familiarity with graphical conventions (Bartlett and Camba 2023b). To examine whether the items from these two tests measured distinct abilities, we compared a unidimensional 2PL model (Model A, Figure 3), indicating that all 38 items measure a common dimension of ability, to a non-hierarchical multidimensional 2PL model (Model B, Figure 3), indicating that the two tests measure distinct but correlated constructs. In the non-hierarchical multidimensional (two-factor) 2PL model (Model B) items from the SBST were constrained to load on one factor, and items from the PRT were constrained to load on a second factor. The unidimensional 2PL model (Model A) showed an acceptable fit to the observed responses (see Table 8). The overall fit of the two-factor model (Model B) was poorer than for Model A (see Table 8), supporting the idea that the tests were measuring the same, rather than distinct abilities, despite the differences in depth cues provided. In the unidimensional model, all items showed a good fit (*p* > .06). The least discriminable items were PRT 11 (discriminability = 0.298) and PRT 10 (discriminability = 0.410). Items functioned well over a wide range of abilities (thetas = −2 to 2). The overall test captured the most information around average to just above average ability but also captured a good amount from moderately low ability (theta = −1) to high ability (theta = 2) levels (see Figure 5). This test showed high factor saturation (McDonald’s Omega = 0.86) and high reliability (split-half reliability = 0.83; Cronbach’s Alpha = 0.85).

Previous research indicated that both the orientation of the cutting plane and the complexity of the solid to be sliced affect the difficulty of items on the SBST (Cohen and Hegarty 2007). To examine whether these item characteristics defined sub-factors, we evaluated a hierarchical bifactor model (Model C, see Figure 2). Sometimes referred to as a two-tier model, ‘bifactor’ refers to the general factor *g* that all items are assumed to load on (i.e., the second tier) and the first tier, which can contain any number of sub-factors, up to the number of items on the test (Gibbons and Hedeker 1992; Chalmers 2012). Model C assumed each item to load on a general factor (cross-section ability) but was also constrained to load on one of four sub-factors, identified based on Cohen and Hegarty’s (2007) classification. Figures were classified as either *simple* or *complex*, and the orientation of the intersecting plane was classified as either *orthogonal* or *oblique* (relative to the x, y, or z-axis of the figure), resulting in four possible sub-factors (7 *simple orthogonal*; *11 complex orthogonal*; *8 simple oblique*; and *12 complex oblique*). The bifactor model showed a very good fit to the observed data (see Table 8), with a better fit than Model A or B, indicating that the 38-item hybrid test is measuring one general factor of cross-section ability, but responses are also influenced by sub-factors of each item.

Finally, we considered more parsimonious bifactor models that specified only the orientation of the intersecting plane (Model D) and only the complexity of the figure (Model E) as sub-factors. As shown in Table 8, Model D fit the observed data significantly better than Model C (AIC and BIC decreased by 80.37, chi-square = 80.37, *p* < .001). In contrast, Model E was a poorer fit than Model C (AIC and BIC increased by 25.28, chi-square = 25.28, *p* < .001) and Model D (*p* < .001, Vuong’s test of non-nested models). Model D fits significantly better than Model A, *p* < .001, using Vuong’s test of non-nested models. These analyses and the principle of parsimony indicate that the best-fitting model was one with a general factor for cross-sectioning reflecting items from both the SBST and the PR, with sub-factors for items with oblique and orthogonal cutting planes. See Supplementary Table S3 for all Model D item parameters^4^. 

### 6.10. Individual Differences

The 38-item refined test was used to examine the effect of demographic variables on cross-sectioning ability (see Figure 7). We examined this using both the summed score of the 38 items and Theta estimates (ability scores) from Model D. As shown in Table 9, correlations with the demographic variables were almost identical for the two measures, so we used summed scores for these analyses (the significance of the correlations with sex were marginal, reaching significance in one case). There was no significant sex difference in the score (males’ mean score = 15.73, *SD* = 7.14, females’ mean score = 14.53, *SD* = 6.47; *p* = .05, Cohen’s *d* = 0.18). There was no significant ethnicity difference (Hispanics’ mean score = 14.96, non-Hispanics’ mean score = 15.17; *p* = .76). A one-way ANOVA tested for differences between educational status groups (in high school, graduated from high school, in college), but no difference was found (*F* (1,496) = 0.31, *p* = .58). Participants whose parents’ education was higher (college degree or above) had significantly higher scores (*M* = 16.52, *SD* = 7.31) than those whose parents’ education was lower (*M* = 14.17, *SD* = 6.31; *p* < .001, *d* = 0.35), and there was a significant, positive correlation between score and reported number of math courses taken (*r* = 0.24, *p* < .001) and Word Sum score (*r* = 0.39, *p* < .001). Age (18–20) was not significantly correlated with score but was correlated with educational level (*r* = 0.11, *p* < 0.01), probably because older participants were more likely to have more education. 

As the demographic variables were somewhat correlated (see Table 9), we conducted a regression analysis with performance (total score) as the outcome variable and the following predictors: sex (male = 0, female = 1), age (18–20), number of math courses taken (1–10), parents’ education (1–5, 1 = did not finish high school, 5 = graduate or professional degree), current education status (1–3, 1 = in high school, 2 = graduated from high school, 3 = in college), and verbal ability (Word Sum score). All predictors except sex were standardized. The results of the regression model using summed scores as the measure of ability are shown in Table 10 (results of the model using theta as the ability measure are in supplementary materials). When accounting for all demographic variables, only the number of math courses and Word Sum score significantly explain variance in performance. The total variance explained using the model is R^2^ = 0.19, *F*(6, 491) = 18.79, *p* < 0.01. 

### 6.11. Creating A Refined, Efficient Test

In order to create an efficient test of cross-section ability, 20 items were selected from the combined 38-item based on item discrimination and difficulty coefficients in Model D. Of the 20 items, 15 were from SBST, and 5 were from PRT, with 14 orthogonal-plane items and 6 obliques. Since test information on the 38-item test was more precise for higher-ability than lower-ability participants, items with the lowest discrimination (range: 0.392–0.909) that also had average to high difficulty (range: −1.748 to 0.190) were removed. There were four items that had lower discriminability (range: 0.630–0.807) but were not removed in order to balance the number of lower difficulty items (range: 0.325–1.305). The average discriminability of the items in the 20-item test was 1.39 (moderately high), and the average difficulty was 0.04 (average). The range of discriminability of the 20 items was 0.538 (low) to 2.802 (very high), and the difficulty ranged from −2.183 (very difficult) to 3.110 (very easy). The internal reliability of the 20 items was high (split-half reliability = 0.78, McDonald’s Omega = 0.84). The Bifactor model with just the refined 20 items using two factors (orthogonal plane or oblique plane, referred to as model 20-D in the table) showed a very good fit to the observed data (see Table 8).

## 7. Discussion

The goals of this study were to evaluate current tests of crossing section ability using modern psychometric techniques, create an efficient test of this ability that is usable across the ability range of the general population, examine possible subcomponents of this ability, and conduct preliminary analyses of how it is related to demographic variables, including sex, ethnicity, and education.

First, in order to collect data from a large sample, several commonly used paper-and-pencil tests of cross-section ability were adapted for online administration. This involved adapting the tests so that one item was shown at a time and time limits were per item (based on piloting in Study 1) rather than for the whole test. This also ensured that we collected data on all (or most) items from all participants, as is necessary for Item Response Theory analyses. Online administration (via Qualtrics Panels) also allowed us to collect a sample that is more representative of the broader population than typical convenience samples of college students. Participants were 18-to−20-year-olds, recruited to match the demographics of Naval recruits, and included participants still in high school, enrolled in college, and graduated from high school without being enrolled in further education. This enabled us to develop a test that can be used to identify individuals who have the potential to excel in both technical careers and STEM education. 

One goal of our analyses was to provide information on the psychometric properties of existing tests. Our analyses indicated that while the tests were correlated, the Santa Barbara Solids Test (SBST) and the Planes of Reference Test (PRT) had good psychometric properties, while the Crystal Slicing Test (CST) did not. Moreover, the CST test was difficult, and another test, the Geological Block Cross-Section Test (GBCST), was eliminated from consideration after Study 1 as it was deemed too difficult for college students. Both the CST and the GBST were developed for studies involving Geology students (Ormand et al. 2014, 2017), so it is likely that they measure geology knowledge in addition to cross-sectioning ability (analogous to other domain-specific tests such as the Tooth Cross-Section test, Hegarty et al. 2009). However, their correlations with the SBST and PRT indicate that they also measure a more general ability to imagine cross-sections of three-dimensional structures in addition to domain knowledge. The domain-general cross-section test developed here can be used to identify students who are likely to benefit from specific training on cross-sectioning before taking advanced classes in STEM-related disciplines such as geology and anatomy that depend on this skill. More domain-specific cross-section tests might then be used to evaluate their performance in the relevant classes. 

The Planes of Reference and Santa Barbara Solids Tests differed in the provision of depth cues to depict the structure of three-dimensional solids in the two dimensions of the printed page. Our analyses indicated that they appeared to measure the same construct, such that a model indicating a single ability underlying these two tests (Model A) was a better fit to the data than a model (Model B), assuming that they measured distinct abilities. Although it has recently been argued that the interpretation of impoverished graphics (with limited depth cues) may be a source of difficulty in spatial ability measures (Bartlett and Camba 2023b), it appears that the difference in depth cues provided by these two tests is not a major source of variance. That is, although these two tests differ in the depth cues provided, they measure a common ability. 

Cohen and Hegarty (2007) proposed two characteristics of cross-section items that might affect their difficulty: the orientation of the cutting plane and the complexity of the figure. But, they found that only the orientation of the cutting plane had an effect. Here, we examined whether these characteristics defined different sub-factors of the cross-sectioning ability and found that the data were best fit by a hierarchical model (Model D) that assumes a general factor and sub-factors reflecting the orientation of the cutting plane (orthogonal or oblique). Consistent with previous research (Cohen and Hegarty 2007) participants made fewer errors on items on orthogonal sections (mean correct = 0.46, *SD* = 0.20) than oblique (mean correct = 0.32, *SD* = 0.19; *t* = 19.37, *p* < 0.001). We speculate that these items were easier because people have more experience viewing near-orthogonal cross-sections in everyday life (e.g., when slicing vegetables) because they tend to assume that a 2D shape extends orthogonally into 3D space, even for oblique sections (Gagnier and Shipley 2016) or because orthogonal sections often require just recognizing the shape of the resulting cross-section (e.g., a square) rather than identifying metric properties of a shape (e.g., a more or less eccentric rectangle), as suggested by Tsutsumi (2004). Interventions to train cross-sectioning ability might use orthogonal cross-sections to scaffold the ability to imagine more difficult oblique cross-sections. 

Sex differences are found in some spatial tests (e.g., mental rotation) but not others (Linn and Petersen 1985; Voyer et al. 1995). Because mental rotation is the most commonly used measure of spatial ability (Hegarty 2018; Bartlett and Camba 2023a), this may overemphasize the existence and size of sex differences in spatial ability, and it is important to document the size of any sex differences when validating measures. Although close to significance, there was no sex difference in ability found in this study, and the effect size was small (*d* = 0.18) compared to the effects commonly found in tests of mental rotation (*d* = 0.67, Voyer et al. 1995). More generally, a moderate effect of parental education level (*d* = 0.35) and a small correlation (*r* = 0.24) with the number of math courses taken suggest that cross-section ability is somewhat influenced by educational opportunities and other socioeconomic-related experiences. In contrast, ethnicity and current educational status (high school, graduated from high school, or in college) had no effects on performance. When all demographic variables were entered in the same linear model, only math courses and verbal ability predicted performance in the refined measure of cross-sectioning. Importantly, there was no significant effect of sex in this model, indicating no appreciable sex differences in this spatial thinking task. 

We set out to create an efficient cross-section measure that provides accurate and precise measurement across a wide range of abilities. Using the discriminability and difficulty estimates of each item in the best-fitting IRT model (see Model D above), we eliminated items that contributed less information, resulting in a 20-item hybrid test containing items from Santa Barbara Solids and Planes of Reference. Based on the response times in this study, participants should be able to complete the shortened test in under 10 min, including instructions. The test was most accurate for assessing participants close to average ability and within two standard deviations above or below average. As such, this test should be useful for assessing the cross-section ability of most of the population. 

### Limitations and Future Directions

Online data collection presents a series of challenges for maintaining a high level of data quality. Several steps were taken to ensure that participants were making a good effort on the tests; however, as they were administered online, there is no way to know if less well-performing participants understood the instructions or made their best effort. With paid survey-takers, there was concern that some participants would quickly skip through the tests, indicating a lack of effort. It is a known problem that online panels include “superworkers” who make up a very small percentage of all workers but complete a large percentage of available tasks (Robinson et al. 2019), and Qualtrics Panels does not make it possible to select only naïve participants. We identified a subset of participants who responded very quickly on most tests and had close-to-chance performance, but rerunning the analyses without these participants did not change the models appreciably. These issues might be mitigated in future studies by including more manipulation checks to detect unmotivated participants, by giving participants feedback on their performance, which may motivate them to try their best (Condon and Revelle 2014), or by gamifying the tests (Malanchini et al. 2020). Time limits were determined using pilot data. However, response times were not analyzed for each item. Likely, more difficult items would require more time, and an item-level analysis of response times could serve to further refine these tests, but that is beyond the scope of this study. In future research, it will also be important to validate this measure against success in both technical occupations and STEM disciplines. 

## 8. Conclusions

This paper introduces and illustrates an approach to developing efficient and robust measures of spatial ability that are normed for the general population. Using this method, we determined that current tests of cross-sectioning were valid in the sense that they explained common variance and that the model assuming a general cross-section factor was a good fit. The Item Response Theory also enabled us to identify the most discriminating and informative items on these measures, enabling us to construct a shorter efficient test that measures performance across the ability range of US high school graduates. This approach can be applied to developing robust measures of other STEM-relevant spatial skills for selection and measuring the effects of educational and training interventions.

## Figures and Tables

**Table 1:** Descriptive statistics for the four measures included in the pilot study.

Test	Possible Range	Score		Response Time		McDonald’s Omega	Spearman- Brown	Cronbach’s Alpha
		*M*	*SD*	*M*	*SD*			
Santa Barbara Solids	0–30	19.32	6.71	11.49	6.04	0.91	0.88	0.89
Planes of Reference	0–15	7.30	3.00	13.76	11.41	0.74	0.63	0.66
Crystal Slicing	0–15	8.26	2.82	12.13	7.50	0.73	0.57	0.64
Geologic Block Cross-Sectioning	0–16	5.77	2.90	16.63	10.77	0.74	0.60	0.64

**Table 2:** Correlation matrix for the measures used in the pilot study. Disattenuated correlations (corrected for reliability of each measure) are shown in parentheses; 95% confidence intervals of the correlations are shown above the diagonal.

	Santa Barbara Solids	Planes of Reference	Crystal Slicing	Geologic Block Cross-Sectioning
Santa Barbara Solids Test	1	[0.60, 0.87]	[0.69, 0.90]	[0.31, 0.74]
Planes of Reference Test	0.76 (1)	1	[0.52, 0.83]	[0.31, 0.74]
Crystal Slicing Test	0.82 (1)	0.71 (1)	1	[0.24, 0.70]
Geologic Block Cross-Sectioning Test	0.56 (0.77)	0.56 (0.91)	0.50 (0.86)	1

**Table 3:** Participant demographics.

Race		Ethnicity		Education Status		Parents’ Education	
White	334	Hispanic	132	In High School	78	High School Diploma	226
Black/African American	106	Not Hispanic	366	In 2Year College	129	Associate’s Degree	52
Asian/Asian American	13			In 4-Year College	151	College Degree	110
American Indian/Alaska Native	7			Not in an Educational Institution	140	Graduate/ Professional Degree	90
Not Stated	38						

**Table 4:** Descriptive statistics for the tests in Study 2.

Test	Possible Range	Score		Response Time		McDonald’s Omega	Spearman- Brown	Cronbach’s Alpha
		*M*	*SD*	*M*	*SD*			
Santa Barbara Solids Test	0–30	12.11	5.53	9.42	1.96	0.84	0.79	0.82
Planes of Reference Test	0–15	5.24	2.44	9.96	2.65	0.55	0.46	0.50
Crystal Slicing Test	0–15	5.28	2.55	9.16	2.45	0.63	0.51	0.56
Word Sum Test	0–14	6.21	2.87	-	-	0.74	0.63	0.72

**Table 5:** Correlations between the measures. Numbers in parentheses indicate partial correlations between the cross-section tests after controlling for the Word Sum test. Above the diagonal (in italics) are disattenuated correlations.

Test	SBST	PRT	CST	WST
Santa Barbara Solids Test (SBST)	1	*0.90*	*0.92*	*0.48*
Planes of Reference Test (PRT)	0.54 * (0.48 *)	1	*1*	*0.67*
Crystal Slicing Test (CST)	0.58 * (0.53 *)	0.50 * (0.43 *)	1	*0.61*
Word Sum Test (WST)	0.34 *	0.36 *	0.35 *	1

* *p* < .001.

**Table 6:** Standardized local dependence diagnostics for all models. Extreme values falling far outside the normal distribution indicate possible item pairs showing local dependence.

Model	G^2^		JSI	
	Min	Max	Min	Max
Santa Barbara Solids Test	−0.13	0.14	−1.39	1.39
Planes of Reference Test	−0.08	0.13	−1.41	1.93
Crystal Slicing Test	−0.11	0.16	−1.36	2.28

**Table 7:** Fit of 2PL unidimensional model for the Santa Barbara Solids Test, Planes of Reference Test, and Crystal Slicing Test.

Test	M_2_ Statistic	df(M_2_)	*p*	RMSEA	TLI	CFI	SRMR
Santa Barbara Solids Test	697.52	405	<.001	0.04	0.93	0.94	0.05
Planes of Reference Test	111.21	90	=.06	0.02	0.94	0.95	0.04
Crystal Slicing Test	220.13	90	<.001	0.05	0.78	0.81	0.06

**Table 8:** Model fit indices for Models A, B, C, D, E, and 20-D.

Model	M_2_	df(M_2_)	*p*	RMSEA	TLI	CFI	SRMR	AIC	BIC
A (Unidimensional 2PL)	1104.01	665	<.001	0.036	0.937	0.941	0.051	21,153.29	21,473.29
B (Multidimensional 2PL)	1275.63	663	<.001	0.043	0.912	0.917	0.094	21,359.02	21,687.45
C (Hierarchical Bifactor, 4 Sub-factors)	860.90	627	<.001	0.027	0.965	0.968	0.046	21,047.96	21,527.97
D (Hierarchical Bifactor, 2 Sub-factors, Orientation)	775.37	627	<.001	0.022	0.978	0.980	0.042	20,967.59	21,447.60
E (Hierarchical Bifactor, 2 Sub-factors, Complexity)	923.84	627	<.001	0.031	0.955	0.960	0.047	21,073.25	21,553.25
20-D (20-item version of D)	207.49	150	=.001	0.028	0.981	0.985	0.039	10,810.84	11,063.47

**Table 9:** Correlation between demographic variables, education, verbal ability, total score on the 38-item test, and theta scores from Model D.

	Sex	Age	Education	Parents’ Education	Math Courses	Word Sum	Total Test Score
Age	0.07						
Education	0.09	0.11 **					
Parents’ Education	−0.03	0.02	0.36 **				
Math Courses	−0.01	0.01	0.17 **	0.20 **			
Word Sum	−0.02	0.02	0.09 *	0.17 **	0.28 **		
Total Test Score	−0.09	−0.07	0.07	0.16 **	0.24 **	0.39 **	
Theta	−0.09*	−0.08	0.07	0.16 **	0.25 **	0.39 **	0.96 **

Note: * *p* < 0.05, ** *p* < 0.01.

**Table 10:** Regression coefficients for linear model with demographic variables as predictors of score.

	Estimate	SE	*t* Value	*p* Value
Intercept	16.60	0.89	18.61	<0.01
Sex	−0.98	0.56	−1.75	0.08
Age	−0.50	0.28	−1.78	0.08
Math Courses	0.90	0.29	3.04	<0.01
Parents’ Education	0.48	0.30	1.58	0.12
Education Status	0.04	0.30	0.15	0.88
Word Sum Score	2.32	0.29	7.94	<0.01

## Data Availability

We are working on data curation and will make all resources available online following the review process.

## Notes

1 We did not include the original Mental Cutting test, because it is identical in format to the Planes of Reference test, or the Tooth tion test, as a previous study indicated that it depended on dentistry expertise (Hegarty et al. 2007).

2 The research was funded by the Office of Naval Research, which is interested in the spatial abilities of potential Navy recruits. The demographics of the US Navy are similar to that of the whole country, cf. Census Bereau (2022) and Department of Defense (2017).

3 Although Hu and Bentler’s (1999) recommended cutoffs for these indices may be too low for IRT, they are used as a rough heuristic here as there is not a universally agreed upon value. Cai et al. (2023) recommend 0.97 for the TLI.

4 Analysis of local independence diagnostics indicated no locally dependent item pairs for any of the models except for Model B (which had a right-skewed distribution of the standardized G2 statistic; min = −0.12, max = 0.40). It is possible that removing one item from each of the item pairs with an extreme G2 would improve model fit; however, other models having good fit without removing further items was taken as sufficient evidence that Model B was not the best fit.

## Supplementary Materials

The following supporting information can be downloaded at: https://www.mdpi.com/article/10.3390/jintelligence11110205/s1, Table S1: Item parameters from unidimensional 2PL model (Santa Barbara Solids Test); Table S2: Item parameters from unidimensional 2PL model (Planes of Reference Test); Table S3: Item parameters from hierarchical bifactor model (38-item combined test); Table S4. Correlations between the Theta scores from the unidimensional 2PL model for each measure (and total score for Word Sum Test).
