# Supplementary material for: Visualizing Cross-Sections of 3D Objects: Developing Efficient Measures Using Item Response Theory
Source: J Intell. 2023 Oct 28;11(11):205. doi: 10.3390/jintelligence11110205 (PMC10672093; doi:10.3390/jintelligence11110205)
Supplement: Supplementary file 1 [file jintelligence-11-00205-s001.zip › jintelligence-2372491-supplementary.pdf]

Journal of *Intelligence*

Article

Visualizing Cross Sections of 3D Objects: Developing Efficient Measures Using Item Response Theory

Table S1. Item Parameters from Unidimensional 2PL Model (Santa Barbara Solids Test)

Item	Discriminability	Difficulty
SBST 1	2.59	0.10
SBST 2	0.69	-0.14
SBST 3	0.71	-1.12
SBST4	1.79	-1.34
SBST 5	1.13	1.24
SBST 6	1.26	1.73
SBST 7	0.92	-0.81
SBST 8	0.76	-0.58
SBST 9	0.74	-1.02
SBST 10	1.49	-1.22
SBST 11	0.69	-0.34
SBST 12	0.88	0.86
SBST 13	1.29	-1.05
SBST 14	0.73	1.18
SBST 15	0.68	-1.18
SBST 16	0.25	-0.51
SBST 17	1.11	0.29
SBST 18	0.79	0.19
SBST 19	0.95	0.30
SBST 20	0.67	-1.36
SBST 21	1.54	-0.34
SBST 22	0.93	-1.76
SBST 23	0.83	-1.35
SBST 24	0.99	0.16
SBST 25	1.64	-2.12
SBST 26	0.69	-1.50
SBST 27	0.90	-0.72
SBST 28	0.74	0.78
SBST 29	0.85	-1.60
SBST 30	0.26	-1.57

Table S2. Item Parameters from Unidimensional 2PL Model (Planes of Reference Test)

Item	Discriminability	Difficulty
PRT 1	2.45	-0.95
PRT 2	1.11	0.29
PRT 3	1.20	-0.83
PRT 4	1.55	-2.45
PRT 5	0.08	-1.24
PRT 6	0.22	-1.03
PRT 7	-0.08	0.14
PRT 8	1.00	0.13

PRT 9	0.26	-0.41
PRT 10	0.51	-2.01
PRT 11	0.35	-0.54
PRT 12	-0.01	-1.06
PRT 13	0.57	-0.16
PRT 14	0.56	-0.62
PRT 15	0.43	-0.93

Table S3. Item Parameters from Hierarchical Bifactor Model (Model D) 38-Item Combined Test

Item	General factor	Sub-factor 1 (orthogonal)	Sub-factor 2 (oblique)	Difficulty
SBST 1*	2.64	0.19	0	0.08
SBST 2	0.56	0.56	0	-0.15
SBST 3	0.77	0	0.34	-1.16
SBST4*	2.01	-0.29	0	-1.46
SBST 5*	0.98	0.77	0	1.29
SBST 6*	1.50	2.43	0	2.75
SBST 7	0.91	0	-0.02	-0.81
SBST 8	0.70	0	0.35	-0.59
SBST 9	0.75	0	0.35	-1.04
SBST 10*	1.89	0	-0.41	-1.42
SBST 11	0.58	0.73	0	-0.36
SBST 12*	0.80	0.73	0	0.91
SBST 13*	1.21	0.28	0	-1.04
SBST 14*	0.63	0.87	0	1.31
SBST 15	0.59	0	0.77	-1.28
SBST 17*	1.06	0.25	0	0.28
SBST 18	0.74	0.42	0	0.19
SBST 19*	0.81	0.97	0	0.33
SBST 20	0.59	0	0.37	-1.37
SBST 21*	1.93	-0.21	0	-0.40
SBST 22*	0.91	0	0.49	-1.81
SBST 23	0.81	0	0.40	-1.38
SBST 24	0.89	0.78	0	0.17
SBST 25*	1.72	0	1.03	-2.35
SBST 26	0.69	0	1.86	-2.19
SBST 27*	0.94	0	-0.08	-0.74
SBST 28*	0.72	0.21	0	0.79
SBST 29	0.81	0	0.84	-1.75
PRT 1*	2.11	-0.61	0	-0.87
PRT 2*	0.97	0.01	0	0.28
PRT 3*	0.97	0	-0.15	-0.77
PRT 4*	1.48	-0.55	0	-2.47
PRT 8*	1.06	0	-0.45	0.14
PRT 10	0.61	0	-0.33	-2.09
PRT 11	0.39	0	-0.08	-0.55
PRT 13	0.46	0	-0.22	-0.16
PRT 14	0.51	0	0.06	-0.61
PRT 15	0.42	0	0.35	-0.95

*Items selected for 20-item test

Table S4. Correlations between the Theta scores from the unidimensional 2PL model for each measure (and total score for Word Sum Test). Above the diagonal is shown the partial correlations between Theta scores controlling for Word Sum scores.

Test	SBST	PRT	CST
Santa Barbara Solids Test (SBST)	1	*.56**	*.58**
Planes of Reference Test (PRT)	.61*	1	*.49**
Crystal Slicing Test (CST)	.63*	.55*	1
Word Sum Test (WST)	.36*	.35*	.34*

*$p < .001$

S.X. Individual Differences

The Theta (ability) scores from Model D were used to compare the groups. There was a small but significant sex difference (men, M = .087, and women, M = -.085; t = 2.07, df = 488.58, p = 0.039, Cohen's d = 0.19). There was no significant difference in Theta for Hispanics (M = -0.047) and non-Hispanics (M = .015), (t = -0.670, df = 241.7, p = 0.503). A one-way ANOVA tested for differences between educational status groups (in high school, graduated from high school, in college), but no difference was found (F = 2.164, p = .142). Participants whose parents' education was higher (college degree or above) had significantly higher scores (M = 0.190) than those whose parents' education was lower (M = -.131), (t = 3.7432, df = 385.1, p < 0.001, Cohen's d = .352), and there was a significant, positive correlation between Theta and reported total number of math courses taken (r = .25, p < .001) and Word Sum score (r = 0.39, p < .001). The only difference from the model using summed scores is (see Table 8) that the effect of age is significant.

	Estimate	SE	*t* value	*p* value
Intercept	.21	0.12	1.75	0.08
Sex	-.14	0.08	-1.88	0.06
Age	-0.08	0.04	-2.07	0.04*
Math Courses	0.12	0.04	3.11	<0.01**
Parents' Education	0.06	0.04	1.57	0.12
Education Status	0.00	0.04	0.07	0.94
Word Sum Score	0.32	0.04	8.02	< 0.01**
